# A Cross-Sectional Exploratory Study of Rat Sarcoid (Ras) Activation in Women with and Without Polycystic Ovary Syndrome

**DOI:** 10.3390/cells14050377

**Published:** 2025-03-05

**Authors:** Sara Anjum Niinuma, Haniya Habib, Ashleigh Suzu-Nishio Takemoto, Priya Das, Thozhukat Sathyapalan, Stephen L. Atkin, Alexandra E. Butler

**Affiliations:** 1 Research Department, Royal College of Surgeons of Ireland, Busaiteen, Adliya P.O. Box 15503, Bahrain; 20202566@rcsi-mub.com (S.A.N.); 20200331@rcsi-mub.com (H.H.); 24205013@rcsi-mub.com (A.S.-N.T.); pdas@rcsi.com (P.D.); satkin@rcsi.com (S.L.A.); 2 Academic Endocrinology, Diabetes and Metabolism, Hull York Medical School, Hull HU6 7RX, UK; thozhukat.sathyapalan@hyms.ac.uk

**Keywords:** polycystic ovary syndrome (PCOS), cardiovascular risk, biomarkers, proteomics, rat sarcoid proteins, ras

## Abstract

**Objective**: Rat sarcoma (Ras) proteins, Kirsten, Harvey, and Neuroblastoma rat sarcoma viral oncogene homolog (KRAS, HRAS, and NRAS, respectively), are a family of GTPases, which are key regulators of cellular growth, differentiation, and apoptosis through signal transduction pathways modulated by growth factors that have been recognized to be dysregulated in PCOS. This study explores Ras signaling proteins and growth factor-related proteins in polycystic ovary syndrome (PCOS). **Methods**: In a well-validated PCOS database of 147 PCOS and 97 control women, plasma was batch analyzed using Somascan proteomic analysis for circulating KRas, Ras GTPase-activating protein-1 (RASA1), and 45 growth factor-related proteins. The cohort was subsequently stratified for BMI (body mass index), testosterone, and insulin resistance (HOMA-IR) for subset analysis. **Results**: Circulating KRas, and RASA1 did not differ between PCOS and control women (*p* > 0.05). EGF1, EGFR, and EGFRvIII were decreased in PCOS (*p* = 0.04, *p* = 0.04 and *p* < 0.001, respectively). FGF8, FGF9, and FGF17 were increased in PCOS (*p* = 0.02, *p* = 0.03 and *p* = 0.04, respectively), and FGFR1 was decreased in PCOS (*p* < 0.001). VEGF-D (*p* < 0.001), IGF1 (*p* < 0.001), IGF-1sR (*p* = 0.02), and PDGFRA (*p* < 0.001) were decreased in PCOS compared to controls. After stratifying for BMI ≤ 29.9 kg/m^2^, EGFR FGF8, FGFR1 VEGF-D, IGF1, and IGF-1sR differed (*p* < 0.05) though EGF1, EGFRvIII, FGF8, FGFR1, and VEGF-D no longer differed; after subsequently stratifying for HOMA-IR, only FGFR1, VEGF-D, IGF1, and IGF-1sR differed between groups (*p* < 0.05). **Conclusions**: Several growth factors that activate Ras differ between women with and without PCOS, and when stratified for BMI and HOMA-IR, only FGFR1, VEGF-D, IGF1, and IGF-1sR differed; these appear to be inherent features of the pathophysiology of PCOS.

## 1. Introduction

Menstrual irregularity, anovulatory infertility and hirsutism are classic features of polycystic ovary syndrome (PCOS) which is a multifactorial endocrine disorder affecting approximately 5–20% of women of reproductive age worldwide [1]. It is recognized that there is an increase in the prevalence of metabolic features including type 2 diabetes (T2D), hypertension, and cardiovascular disease in PCOS (1). However, the inherent mechanism is still unclear, insulin resistance (IR) and obesity-related inflammation associated with PCOS have been implicated [2,3].

Rat sarcoma (Ras) proteins, including Kirsten rat sarcoma viral oncogene homolog (KRAS), Harvey Rat sarcoma virus (HRAS), and neuroblastoma Ras viral oncogene homolog (NRAS), are a family of small GTPases that are key regulators of a wide array of cellular processes that involve growth, differentiation, and apoptosis through their actions in signal transduction pathways [4]. PCOS is often associated with IR, and Ras proteins are involved in the insulin signaling pathway, particularly through the Ras-MAPK (Mitogen-Activated Protein Kinase) pathway [5], which regulates cellular responses such as growth and differentiation [6].

Ras proteins play a role in follicular development and steroidogenesis by mediating signals from gonadotropins such as luteinizing hormone (LH) [7]. Ras-MAPK signaling is involved in the pathways that regulate androgen production in the ovaries and thus may contribute to hyperandrogenism, one of the diagnostic features of PCOS [8].

PCOS is associated with a pro-inflammatory state and Ras proteins are involved in regulating inflammatory responses, with activation of Ras contributing to the chronic low-grade inflammation seen in PCOS patients; KRAS is a potential pharmacological target to treat PCOS [9].

**Figure 1:**
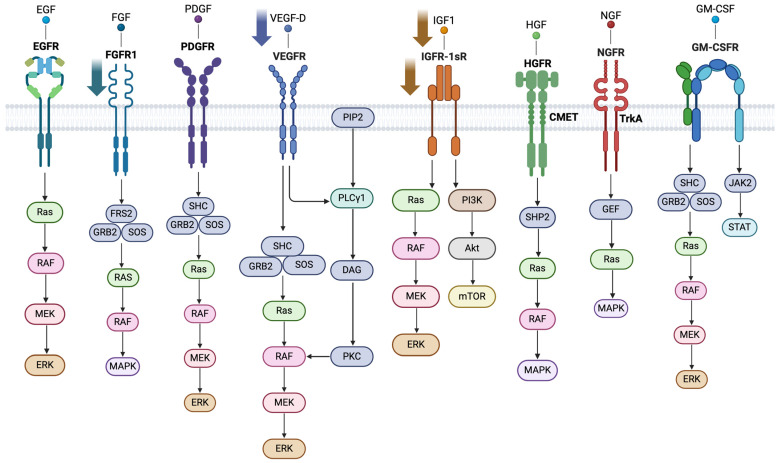
Illustration of growth factors and their activation of Ras. The downward arrows indicate the lower plasma levels found in women with polycystic ovary syndrome (PCOS) independent of BMI, inflammation and insulin resistance (EGF: Epidermal Growth Factor, EFGR: Epidermal Growth Factor Receptor, FGF: Fibroblast Growth Factor, FGFR: Fibroblast Growth Factor Receptor, PDGF: Platelet-derived Growth Factor, PDGFR: Platelet-derived Growth Factor Receptor; VEGF: Vascular Endothelial Growth Factor, VEGFR: Vascular Endothelial Growth Factor Receptor, IGF: Insulin and Insulin-like Growth Factor; IGFR: Insulin and Insulin-like Growth Factor receptor, HGF: Hepatocyte Growth Factor, HGFR: Hepatocyte Growth Factor Receptor, C-MET: Mesenchymal-Epithelial Transition Factor, NGF: Nerve Growth Factor, NGFR: Nerve Growth Factor Receptor, TrkA: Tropomyosin receptor kinase A, GM-CSF: Granulocyte-Macrophage Colony-Stimulating Factor, GM-CSFR: Granulocyte-Macrophage Colony-Stimulating Factor Receptor, Ras: Rat Sarcoma Virus, RAF: Rapidly Accelerated Fibrosarcoma, MEK: Mitogen-activated Protein Kinase Kinase, ERK: Extracellular Signal-regulated Kinase. MAPK: Ras/mitogen-activated Protein Kinase, FRS2: Fibroblast Growth Factor Receptor Substrate, GRB2: Growth Factor Receptor-bound Protein-2, SOS: Son of Sevenless, SHC: Src Homology and Collagen, PIP2: Phosphatidylinositol 4,5-bisphosphate, PLCγ: Phospholipase C Gamma, DAG: Diacylglycerol, PKC: Protein Kinase C, PIK3: Phosphoinositide 3-Kinase, Akt: Protein Kinase B, mTOR: Mammalian Target of Rapamycin, SHP2: Src Homology 2 Domain Containing Phosphatase 2, JAK2: Janus Kinase 2, STAT: Signal Transducer and Activator of Transcription).

Circulating Ras proteins, whether as fragments, exosome-associated proteins or Ras-related signaling components, are found in cancer and metabolic disorders [10]; however, circulatory Ras protein levels have not previously been reported in PCOS. Circulating growth factors that act through the Ras signaling system do, however, differ in PCOS, examples of which include Epidermal growth factor (EGF), which binds to the EGF Receptor (EGFR, also known as ErbB1) [11,12]. Fibroblast growth factors (FGFs), which bind to FGF Receptors (FGFRs), affect ovarian function in PCOS [13]. Platelet-derived growth factor (PDGF), binding to the PDGF Receptor (PDGFR), may modulate steroid production involved in fertility [14] in PCOS. Vascular endothelial growth factor (VEGF), binding to the VEGF Receptor (VEGFR), plays a critical role in angiogenesis and ovarian folliculogenesis, with the suggestion that it is associated with increased PCOS risk [15]. Insulin-like growth Factors (IGFs) activate Ras and play an important role in the pathogenesis of PCOS related to insulin resistance and inflammatory responses [16]. Other growth factors acting through Ras include the Hepatocyte growth factor (HGF), binding to the HGF receptor (HGFR), and Nerve growth factor (NGF), binding to the nerve growth factor receptor (NGFR). The growth factors and their activation of Ras are shown in Figure 1.

This study explores Ras signaling in PCOS, with a particular focus on the potential importance of circulatory Ras proteins and growth factors that activate Ras intracellular signaling pathways in women with and without PCOS from a UK Biobank.

## 2. Materials and Methods

### 2.1. Study Design

This study was designed as a cross-sectional analysis, incorporating a total of 234 Caucasian women aged between 18 and 36 years. Among them, 147 were diagnosed with polycystic ovary syndrome (PCOS), while 97 served as healthy controls; all subjects had been recruited to a PCOS biobank (ISRCTN70196169: 2012–2017) based in the Department of Endocrinology, Hull Royal Infirmary, UK, with approval from the Newcastle and North Tyneside Ethics Committee [17]. Each participant provided written informed consent. PCOS diagnosis was established using the Rotterdam criteria [18], requiring the presence of at least two of the following: oligo/anovulation, hyperandrogenism (defined by a Ferriman-Gallwey score > 8, a free androgen index exceeding 4, or total testosterone levels above 1.5 nmol/L, based on local laboratory references), or polycystic ovarian morphology confirmed via transvaginal ultrasound. Secondary conditions such as nonclassical 21-hydroxylase deficiency were excluded through appropriate screening, as previously outlined [17]. The demographic details of both PCOS and control groups are provided in Table 1 [17]. Control participants exhibited regular menstrual cycles, unremarkable physical examinations, and no evidence of polycystic ovaries upon ultrasound assessment. They were also medication-free.

Fasting blood samples were collected at the time of informed consent, centrifuged at 3500× *g* for 15 min, and stored at −80 °C in aliquots. Various biomarkers were analyzed, including sex hormone-binding globulin (SHBG), insulin (measured using a DPC Immulite 200 analyzer, Euro/DPC, Llanberis UK), lipid measurements, and plasma glucose, which were utilized to compute the homeostasis model assessment of insulin resistance (HOMA-IR) (Synchron LX20 analyzer, Beckman-Coulter, High Wycombe, UK). The free androgen index (FAI) was derived by dividing total testosterone by SHBG and multiplying by 100. Serum testosterone levels were determined using isotope-dilution liquid chromatography tandem mass spectrometry (LC-MS/MS) [19] Anti-Müllerian hormone was measured using a Beckman Coulter Access automated immunoassay.

Plasma protein levels were assessed through the Slow Off-rate Modified Aptamer (SOMA)-scan platform [20], which facilitated the quantification of multiple proteins, including GTPase Kirsten rat sarcoma virus (KRAS), Ras GTPase-activating protein 1 (RASA1), Heparin-binding epidermal growth factor-like growth factor (HB-EGF), Epidermal growth factor receptor variant III (EGFRvIII), Epidermal growth factor (EGF), Epidermal growth factor receptor (EGFR), Fibroblast growth factor 8 isoform A (FGF-8A), Fibroblast growth factor 8 isoform B (FGF-8B), Fibroblast growth factor receptors 1–4 (FGFR1, FGFR2, FGFR3, FGFR4), Fibroblast growth factor 2 (FGF2), Fibroblast growth factors 4–7 (FGF4, FGF5, FGF6, FGF7), Fibroblast growth factors 9–10 (FGF9, FGF10), Fibroblast growth factor 12 (FGF12), Fibroblast growth factors 16–20 (FGF16, FGF17, FGF18, FGF19, FGF20), Fibroblast growth factor 23 (FGF23), Platelet-derived growth factor C (PDGFC), Platelet-derived growth factor subunit A (PDGFA), Platelet-derived growth factor subunit B (PDGFB), Platelet-derived growth factor receptor beta (PDGFRB), Platelet-derived growth factor receptor alpha (PDGFRA), Vascular endothelial growth factor A (VEGFA), Vascular endothelial growth factor A isoform 121 (VEGFA-121), Vascular endothelial growth factor C (VEGF-C), Vascular endothelial growth factor D (VEGF-D), Vascular endothelial growth factor receptor 2 (VEGF-sR2), Vascular endothelial growth factor receptor 3 (VEGF-sR3), Insulin (INS), Insulin-like growth factor I (IGF1), Macrophage colony-stimulating factor 1 (CSF1), Macrophage colony-stimulating factor 1 receptor (CSF1R), Granulocyte-macrophage colony-stimulating factor 2 (CSF2), Granulocyte colony-stimulating factor (CSF3), Granulocyte colony-stimulating factor receptor (CSF3R), Insulin-like growth factor 1 receptor (IGF1R), Cation-independent mannose-6-phosphate receptor (IGF2R), Hepatocyte growth factor (HGF), and beta-nerve growth factor (NGF). Calibration was based on standards as previously described [21].

Protein quantification was performed using an aptamer-based approach known as the SOMAmer protein array [22,23]. Plasma samples collected in EDTA tubes underwent a structured protocol: (1) SOMAmers bound to analytes using a photocleavable linker; (2) The complexes were immobilized on a streptavidin-coated surface; (3) Ultraviolet (UV) exposure released analyte-SOMAmer complexes into solution; (4) These complexes were immobilized once more through biotin-streptavidin interactions; (5) Eluted SOMAmers served as surrogates for analyte quantification; (6) Hybridization to complementary oligonucleotides enabled final quantification. Normalization and standardization of raw intensities, hybridization signals, medians, and calibration data followed established methodologies [20,21].

### 2.2. Statistics

Continuous variables were presented as means ± standard deviations (SD). To compare circulating levels of KRAS, Ras GTPase-activating protein-1 (RASA1), and 45 growth factor-related proteins between the PCOS and control groups, independent two-sample *t*-tests were employed. A significance threshold of *p* < 0.05 was applied. The quantile normalized SOMAscan proteomic data was log-transformed for further statistical assessments. Linear models for microarray (limma) analysis in conjunction with *t*-tests were used to compare and identify the dysregulated proteins in PCOS versus controls. Any protein changes with a fold change of 1 and raw *p*-value < 0.05 were considered significant. Spearman’s rank correlations were computed to examine associations between growth factor-related proteins and body mass index (BMI). Statistical analyses were conducted using RStudio (version 2023.03.0), with all tests performed as two-tailed analyses, considering *p*-values below 0.05 as statistically significant.

## 3. Results

Baseline data for the 147 PCOS subjects and 97 controls are shown in Table 1. The two cohorts were age-matched, but subjects with PCOS had a greater BMI, increased IR, hyperandrogenemia, increased C-reactive protein (CRP, an inflammatory marker), and a raised anti-Mullerian hormone. PCOS subjects had higher systolic and diastolic blood pressures, a higher fasting blood glucose, and a lower high-density lipoprotein (Table 1, Supplementary Figure S1).

**Figure 2:**
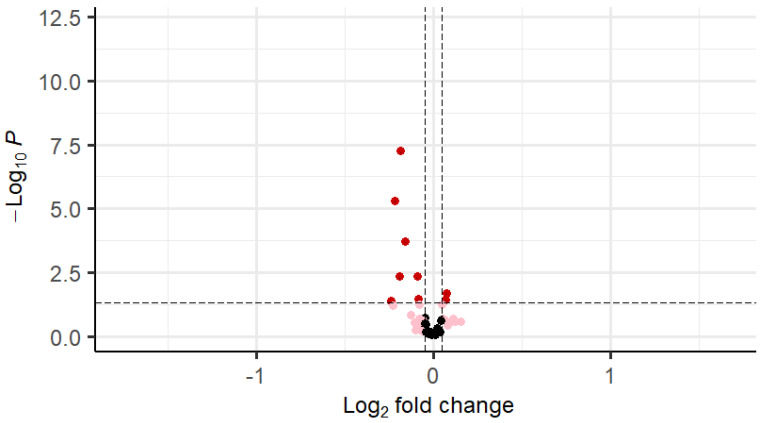
Volcano plot for differentially regulated genes in whole cohort (the black dots denote the non-significant genes, pink dots denote the genes with fold change of 1 and raw *p*-value > 0.05, and the red dots indicate the genes with fold change of 1 and raw *p*-value < 0.05.

The results of the Somascan analysis of those proteins that differed between PCOS and control women are shown in Table 2, and all proteins are shown in Supplemental Table S1. Circulating KRas and RASA1 did not differ between PCOS and control women (*p* > 0.05). EGF1, EGFR, and EGFRvIII were decreased in PCOS (*p* = 0.04, *p* = 0.04, and *p* < 0.001, respectively). FGF8, FGF9, and FGF17 were increased in PCOS (*p* = 0.02, *p* = 0.03 and *p* = 0.04, respectively), and FGFR1 was decreased in PCOS (*p* < 0.001). VEGF-D (*p* < 0.001), IGF1 (*p* < 0.001), IGF-1sR (*p* = 0.02), and PDGFRA (*p* < 0.001) were decreased in PCOS compared to controls, as shown in Table 2 and Figure 2.

Fibroblast growth factor receptor 1 (FGFR1; bFGF-R); Insulin-like growth factor I (IGF-1); Vascular endothelial growth factor D (VEGF-D); Platelet-derived growth factor receptor alpha (PDGFRA); Epidermal growth factor receptor variant III (EGFRvIII); Fibroblast growth factor 8 isoform A (FGF-8A); Insulin-like growth factor 1 receptor (IGF-I sR); Fibroblast growth factor 9 (FGF9); Epidermal growth factor receptor (EGFR); Epidermal growth factor (EGF); Fibroblast growth factor 17 (FGF-17); Fibroblast growth factor 19 (FGF-19).

**Figure 3:**
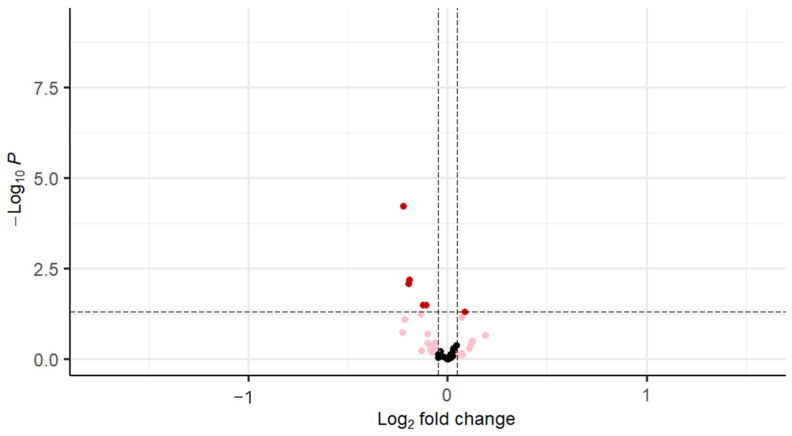
Volcano plot for differentially regulated genes in on BMI matched (BMI ≤ 29.9 kg/m^2^) cohort (the black dots denote the non-significant genes, pink dots denote the genes with fold change of 1 and raw *p*-value > 0.05, and the red dots indicate the genes with fold change of 1 and raw *p*-value < 0.05.

With stratification of the cohort based on BMI (≤29.9 kg/m^2^), EGFR was decreased in PCOS (*p* < 0.03), FGF8 was increased in PCOS (*p* < 0.04), and FGFR1 was decreased in PCOS (*p* < 0.001). VEGF-D (*p* < 0.01), IGF1 (*p* < 0.01), and IGF-1sR (*p* < 0.03) were decreased in PCOS compared to controls, as shown in Table 3 and Figure 3.

Fibroblast growth factor receptor 1 (FGFR1; bFGF-R); Insulin-like growth factor I (IGF-1); Vascular endothelial growth factor D (VEGF-D); Insulin-like growth factor 1 receptor (IGF-I sR); Epidermal growth factor receptor (EGFR); Fibroblast growth factor 8 isoform B (*FGF-8B*).

**Figure 4:**
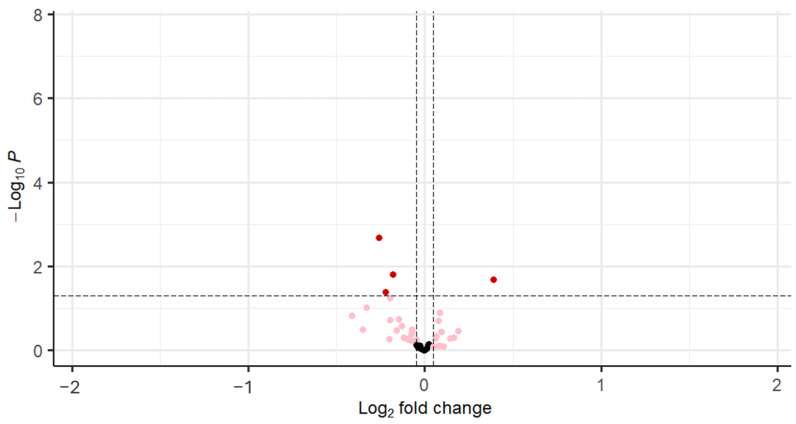
Volcano plot for differentially regulated genes in on BMI (BMI ≤ 29.9 kg/m^2^) and IR (HOMA-IR ≤ 1.9) matched cohort (the black dots denote the non-significant genes, pink dots denote the genes with fold change of 1 and raw *p*-value > 0.05, and the red dots indicate the genes with fold change of 1 and raw *p*-value < 0.05.

With stratification of the cohort based on BMI (≤29.9 kg/m^2^) and HOMA-IR, FGFR1 was decreased in PCOS (*p* < 0.001), and VEGF-D (*p* < 0.04), IGF1 (*p* < 0.04), and IGF-1sR (*p* < 0.02) were decreased in PCOS compared to controls, as shown in Table 4 and Figure 4.

Fibroblast growth factor receptor 1 (FGFR1; bFGF-R); Insulin-like growth factor 1 receptor (IGF-I sR); Insulin-like growth factor I (IGF-1); Vascular endothelial growth factor D (VEGF-D).

**Figure 5:**
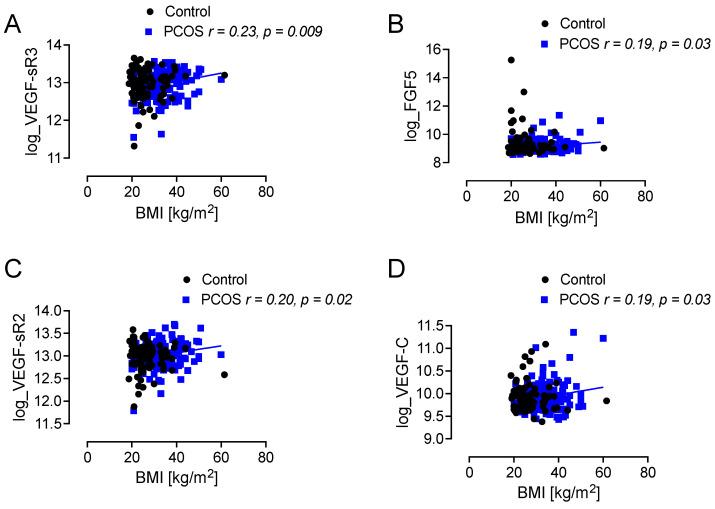
Correlations of body mass index (BMI) with Ras-related proteins. BMI was associated positively with VEGF-sR3 (**A**), FGF5 (**B**), VEGF-sR2 (**C**), and VEGF-C (**D**) only in women with polycystic ovary syndrome (PCOS).

Correlation analysis of the unstratified groups showed that BMI was associated positively with the altered VEGF-sR3, FGF5, VEGF-sR2, and VEGF-C (r = 0.23, *p* = 0.0109; r = 0.19, *p* = 0.03; r = 0.2, *p* = 0.02; r = 0.19, *p* = 0.03, respectively) only in the PCOS subjects as shown in Figure 5, but there was no correlation with BMI and the growth factor-related proteins that differed between PCOS and controls. Neither was there any correlation with inflammation, as adjudged by CRP, or of IR, as adjudged by the Homeostatic Model Assessment for IR (HOMA-IR), with the growth factor-related proteins that differed between PCOS and controls.

## 4. Discussion

Circulatory Ras protein levels have not previously been reported in women with PCOS, making this study the first to explore their potential role in the pathophysiology of the condition. Prior investigations into Ras proteins in PCOS have focused on their expression in specific tissues rather than in the circulation. For instance, Xu et al. analyzed the gene expression of HRAS, NRAS, and KRAS in subcutaneous adipose tissue and found no significant differences in the expression of these Ras family genes between 22 women with PCOS and 13 controls [24]. However, HRAS, NRAS, and KRAS expression showed positive correlations with testosterone levels, highlighting a potential link to androgenic activity. Interestingly, these genes also demonstrated inverse correlations with metabolic traits such as BMI, fasting glucose and IR, linking Ras proteins to the pathogenesis of PCOS.

This study extends the investigation to circulating Ras proteins, providing a broader perspective on their systemic role in PCOS. Notably, circulating Ras proteins KRas and RASA1 did not differ significantly between women with PCOS and controls, indicating that systemic levels of these key Ras proteins may not be altered in the condition. However, despite the absence of significant differences in circulating Ras protein levels, findings from a drug-hub gene interaction network suggest that Ras-related genes, such as KRAS, may still have therapeutic potential [9]. The network identified KRAS, along with other hub genes like Phosphatase and tensin homolog (PTEN), and MAPK1, as potential targets for treatment, with metformin specifically interacting with both PTEN and KRAS. These insights suggest that targeting these pathways, despite their unaltered expression in the circulation, could open new therapeutic avenues for PCOS management, highlighting the complexity of the condition and the potential for exploring Ras-mediated signaling pathways in treatment development.

Of the 45 growth factor-related proteins analyzed for their role in activating Ras intracellular signaling pathways, only 11 demonstrated significant differences between the two groups. Interestingly, among these, three proteins were increased and eight were decreased in PCOS. These results collectively suggest that Ras-activated pathways are not broadly upregulated in PCOS and, therefore, Ras activation may not play a central role in mediating the pathogenesis or phenotype of the condition.

Among the 11 growth factor-related proteins—EGF1, EGFR, EGFRvIII, FGF8, FGF9, FGF17, FGFR1, VEGF-D, IGF1, IGF-1sR, and PDGFRA—that showed significant differences between women with PCOS and controls, these changes were found to be independent of BMI, inflammation (indicated by the lack of correlation with CRP), or IR, suggesting that these differences are intrinsic to PCOS itself. However, when the cohort was stratified by BMI ≤ 29.9 kg/m^2^, only EGFR FGF8, FGFR1 VEGF-D, IGF1, and IGF-1sR remained significant with a loss of significance for EGF1, EGFRvIII, FGF8, FGFR1, and VEGF-D. This showed that these latter proteins are BMI dependent, and highlights that statistical adjustment for BMI may give different results compared to when cohorts are matched for BMI, as the statistical adjustment may result in an over or under estimate given that BMI and PCOS are so closely linked. When the cohorts were stratified for BMI ≤ 29.9 kg/m^2^ and normal HOMA-IR (≤1.9), FGFR1, VEGF-D, IGF1, and IGF-1sR differed, suggesting that these factors are more likely to be inherently different in PCOS rather than an epiphenomenon of an associated PCOS feature such as BMI or IR. Conversely, EGFR and FGF8 were no longer significant indicating that these are dependent on IR, in accord with the literature for EGFR [25], while FGF8 modulation by insulin resistance is a novel finding.

Our findings show reduced plasma EGF1 and EGFR levels in women with PCOS and, here, plasma EGF was no longer different when BMI was stratified and plasma EGFR was no longer different when BMI and IR were stratified, suggesting that EGF1 is BMI dependent; however, there are no comparable studies looking at serum EGF1 and EGFR in PCOS in the literature, and the effect of BMI on EGF1 and EGFR levels from other studies are discrepant. EGF1 and EGFR were negatively regulated with BMI and IR in adipose tissue [26], whilst, in patients with bipolar disorder, BMI did not affect EGF1 levels [27]; however, in breast cancer patients, there is a positive correlation between BMI and EGFR2 [28]. A further study in participants having normal BMI reported higher EGFR levels in the cumulus granulosa cells of PCOS subjects versus controls, but the serum EGFR levels were not assessed in that study [12]. Studies report that elevated EGF and EGFR expression is found in the granulosa cells and follicular fluid of the ovary in PCOS where EGF may inhibit granulosa cell estrogen synthesis, which is translated into arrest of follicle growth [11,12]. EGFR signaling has been proposed as a therapeutic target, as its inhibition improved ovulatory function, estrous cyclicity, and hormone balance [12]. Further studies are needed to determine the effect of BMI on circulating EGF1 and EGFR levels.

Our study found increased levels of FGF8, FGF9, and FGF17 in PCOS patients, marking a novel observation as these growth factors have not been extensively explored in this context; however, as noted above, FGF8 appears to be related to IR. Research on FGF8’s ovarian functions has demonstrated its critical involvement in folliculogenesis, ovulation, and granulosa cell proliferation, suggesting that altered levels of FGF8 in PCOS could impact ovarian function and reproductive outcomes [13]. FGF8’s role has also been studied in modulating granulosa cell function and its interaction with bone morphogenetic protein (BMP) signaling, which regulates ovarian steroidogenesis [29]. FGF8 suppresses follicle-stimulating hormone (FSH)-induced estradiol production while enhancing BMP-Smad signaling, suggesting it contributes to disrupted oocyte–granulosa cell communication in PCOS [29]. The increased FGF8 levels observed in our study may play a role in altered follicular dynamics and impaired estradiol production in PCOS, potentially representing a target for therapeutic intervention. However, a previous study investigating inflammatory proteins in non-obese, non-insulin-resistant PCOS women found no significant difference in FGF8 levels between PCOS and controls, suggesting that the role of FGF8 might vary depending on population characteristics, particularly IR [30].

Conversely, FGF9 regulates granulosa cell function by inhibiting steroid hormone production and specific gene expressions, such as FSH receptor (FSHR) and cytochrome P450 family 11 subfamily A member 1 (CYP11A1), while promoting granulosa cell proliferation [31]. It may also slow follicle development through pathways like IGF-I and cAMP, with hormonal factors such as IGF-I and estradiol influencing FGF9 production, highlighting its role in ovarian function and follicular differentiation [31]. In our study, the increased expression of FGF9 in PCOS participants was lost when stratified for BMI, indicating that it is reflective of obesity rather than inherent to PCOS. FGF9 has been shown to enhance progesterone production in granulosa cells and regulate steroidogenic proteins such as steroidogenic acute regulatory protein (StAR) and cholesterol side-chain cleavage enzyme (P450scc) [32]. Thus, the elevated FGF9 levels in obese PCOS patients could be an attempt to counteract the dysregulation of steroidogenesis and follicular maturation. However, this compensatory effect may be insufficient to overcome the broader metabolic and reproductive challenges characteristic of the condition. However, no studies have specifically investigated FGF9 levels in PCOS compared to control groups, making it challenging to directly compare our findings with existing literature.

FGF17 is detected mainly in oocytes, but also in granulosa cells [33] and there is no literature directly addressing its role in PCOS or metabolism; however, in this study, it can be seen that it appears to be related to BMI as, when BMI was accounted for, it did not differ between women with and without PCOS.

In our study, we observed decreased FGFR1 expression in PCOS, which aligns with findings from another study showing reduced FGFR1 levels in granulosa-lutein cells of women with PCOS [34]. This reduction in FGFR1 may contribute to impaired angiogenesis and follicular development, suggesting a link between FGFR1 dysregulation and ovarian dysfunction in PCOS. Further insights into the role of FGFR1 signaling in reproductive health come from studies indicating that the loss of FGFR1 disrupts cell cycle progression and downregulates key genes such as EZH2, which regulates cell proliferation and differentiation [35]. The decreased expression of FGFR1 in PCOS participants may, therefore, reflect a disruption in cellular pathways that are essential for normal ovarian function and follicle maturation. Interestingly, a recent study has shown that impaired FGFR1 signaling is associated with IR and metabolic dysfunction, further supporting the relevance of our findings [36]; however, after stratification for both BMI and HOMA-IR, it still differed between the women with and without PCOS suggesting that it is independent of both BMI and IR. The decreased expression of FGFR1 in our PCOS participants could reflect a similar disruption in signaling, potentially contributing to the metabolic and reproductive challenges characteristic of the condition.

Additionally, we found decreased levels of VEGF-D expression in PCOS patients, a factor primarily described as a lymphangiogenic molecule. Previous studies have reported significantly elevated concentrations of serum VEGF (also known as VEGF-A) in PCOS patients in comparison to controls [37,38,39,40]. This increase in pro-angiogenic factors like VEGF can contribute to hyperplasia, hypervascularity, and the gradual development of endothelial dysfunction, key features of PCOS [41]. Interestingly, however, our study did not find significant differences in VEGF expression between PCOS patients and controls. Instead, we identified a significant decrease in VEGF-D expression, a less well-researched member of the VEGF family in the context of PCOS. The observed decrease in VEGF-D expression in our study, in contrast to previous findings of elevated VEGF-A levels, may reflect differences in the roles of these VEGF family members in PCOS pathophysiology. Similarly, another study investigating adolescent PCOS patients found no significant differences in VEGF levels between cases and controls, suggesting that VEGF expression may vary across subpopulations and disease stages [42]. Future research should investigate the specific regulatory mechanisms governing VEGF-D expression in PCOS and how these pathways interact with broader angiogenic and lymphangiogenic processes.

We observed decreased levels of IGF1 expression in PCOS patients. This appears to contrast with previous studies that have reported increased serum levels of IGF1 in PCOS patients in comparison to controls [43,44]; however, in a meta-analysis of 20 studies, 5 of these showed higher IGF1 levels in the controls and when stratified for BMI less than or equal to 29 kg/m^2^, IGF1 levels were significantly higher in the control populations, suggesting that the serum IGF1 levels between groups depends on the proportion of subjects with a BMI less than or greater than 29 kg/m^2^ [45]. These results are therefore in accord with the literature when stratifying for BMI in our analysis. In addition, PCOS patients who are obese exhibit reduced growth hormone (GH) secretion, possibly resulting in diminished downstream effects on the regulation of IGF1 [46]. Our findings suggest that IGF1 levels in PCOS are not universally elevated and that the relationship between PCOS and IGF1 dynamics is more complex than previously understood.

PDGFRA was found to decrease in women with PCOS, but after stratifying for BMI, it no longer differed between groups, indicating that it was a feature of obesity rather than PCOS per se. PDGFR signaling is thought to be a common mechanism in the control of multiple steroidogenic lineages involved in fertility [14] and has been suggested as a potential therapeutic target for PCOS [47].

While our findings on growth factor-related proteins provide new insights into the complex hormonal interactions in PCOS, they also underscore the inherent challenges in isolating the effects of PCOS from other confounding factors. Limitations of this study include its focus on a predominantly Caucasian population, which may limit the generalizability of the findings to other ethnic groups. Additionally, while our study sought to examine the role of circulating growth factor-related proteins in PCOS, stratifying for BMI and IR allowed us to determine which factors may be inherent within PCOS. Both BMI and IR are highly correlated with PCOS and its metabolic and hormonal features. As such, regression models adjusting for these factors may show differing results than when covariate adjustment is made up front by accounting for BMI and IR, for instance. Further studies with larger sample sizes and refined statistical approaches, such as stratifying by BMI, insulin sensitivity and systemic inflammation could help address these limitations and provide more definitive insights into the pathways involved in PCOS pathophysiology.

## 5. Conclusions

Several growth factors that activate Ras differ between women with and without PCOS and, when stratified for BMI and HOMA-IR, only FGFR1, VEGF-D, IGF1, and IGF-1sR, differed and those appear to be inherent features of the pathophysiology of PCOS.

## Figures and Tables

**Table 1:** Demographics, baseline hormonal and metabolic parameters of the polycystic ovary syndrome (PCOS) subjects and controls.

Baseline Demographics	PCOS (*n* = 147)	Controls (*n* = 97)
	Mean (SD)	Mean (SD)
Age (years)	29.1 ± 6.1	29.6 ± 6.5
BMI (kg/m^2^)	34.1 ± 7.5	26.7 ± 6.6 ***
Insulin (IU/mL)	10.2 ± 6.1	6.2 ± 3.2 ***
HOMA-IR	3.8 ± 0.6	1.6 ± 0.2 ***
Testosterone (nmol/L)	1.6 ± 1.0	1.05 ± 0.48 ***
SHBG (nmol/L)	42.5 ± 39.6	77.5 ± 78.4 ***
Free androgen index (FAI)	4.5 ± 3.9	2.1 ± 1.4 ***
CRP (mg/L)	4.4 ± 4.2	2.4 ± 3.9 ***
Systolic blood pressure (mmHg)	121 ± 14	114 ± 11 ***
Diastolic blood pressure (mmHg)	77 ± 10	73 ± 11 ***
Fasting blood glucose (mmol/L)	4.9 ± 1.1	4.6 ± 0.6 **
Cholesterol (mmol/L)	4.8 ± 1.0	4.6 ± 0.7
High density lipoprotein (mmol/L)	1.22 ± 0.30	1.43 ± 0.31 ***
Low density lipoprotein 9 mmol/L)	2.90 ± 0.86	2.72 ± 0.6
AMH (ng/mL)	40 ± 31	18 ± 18 ***

BMI—Body Mass Index; HOMA-IR—Homeostasis model of assessment-insulin resistance; CRP—C reactive protein; SHBG—sex hormone binding globulin. ** *p* < 0.01, *** *p* < 0.001.

**Table 2:** Whole set: proteins that differed between PCOS (n = 147) and control (n = 97) women in entire cohort.

Gene	logFC	Average Expression	t	*p* Value
*FGFR1* (bFGF-R)	−0.18	9.36	−5.60	<0.001
*IGF-1*	−0.22	9.58	−4.68	<0.001
*VEGF-D*	−0.16	8.85	−3.79	<0.001
*PDGFRA*	−0.19	9.72	−2.87	<0.001
*EGFRvIII*	−0.09	14.72	−2.86	<0.001
*FGF-8*	0.07	9.53	2.33	0.02
*IGF-I sR*	−0.09	12.49	−2.14	0.02
*FGF9*	0.07	8.93	2.10	0.03
*EGFR*	−0.24	8.25	−2.07	0.04
*EGF*	−0.08	9.34	−1.93	0.04
*FGF-17*	0.05	7.62	1.92	0.04

**Table 3:** Stratification of the cohort based on BMI matched (BMI ≤ 29.9 kg/m^2^): Proteins that differed between PCOS (n = 36) and control (n = 74) women in this BMI matched cohort.

Gene	logFC	Average Expression	t	*p* Value
*FGFR1* (bFGF-R)	−0.22	9.39	−4.17	<0.001
*IGF-1*	−0.19	9.62	−2.77	0.01
*VEGF-D*	−0.20	8.90	−2.70	0.01
*IGF-I sR*	−0.12	12.50	−2.17	0.03
*EGFR*	−0.11	14.74	−2.17	0.03
*FGF-8*	0.08	9.51	1.99	0.04

**Table 4:** Stratification of the cohort based on BMI ≤ 29.9 kg/m^2^ and HOMA-IR ≤ 1.9 matched. Proteins that differed between PCOS (n = 19) and control (n = 47) women for the BMI and IR matched cohort.

Gene	logFC	Average Expression	t	*p* Value
FGFR1 *(bFGF-R)*	−0.26	9.42	−3.20	<0.001
*IGF-I sR*	−0.18	12.49	−2.48	0.02
*IGF-1*	−0.22	9.65	−2.08	0.04
*VEGF-D*	−0.20	8.94	−1.95	0.04

## Data Availability

All the data for this study will be made available upon reasonable request to the corresponding author.

## Supplementary Materials

The following supporting information can be downloaded at https://www.mdpi.com/article/10.3390/cells14050377/s1, Table S1: Differential gene expression analysis results for circulatory Ras proteins and growth factors that activate Ras intracellular signaling pathways in PCOS versus controls; Figure S1: Stratification of Control and PCOS women from the database on BMI (BMI ≤ 29.9 kg/m^2^ and >29.9 kg/m^2^), then stratified by testosterone into non-hyperandrogenic/hyperandrogenic (testosterone 1.5 nmol/L), then stratified by insulin resistance (HOMA 1.9).
