# Supplementary material for: A Cross-Sectional Exploratory Study of Rat Sarcoid (Ras) Activation in Women with and Without Polycystic Ovary Syndrome
Source: Cells. 2025 Mar 5;14(5):377. doi: 10.3390/cells14050377 (PMC11898917; doi:10.3390/cells14050377)

Supplementary Table S1: Differential gene expression analysis results for circulatory Ras proteins and growth factors that activate Ras intracellular signaling pathways in PCOS versus controls.

Gene	logFC	Average Expression	t	P.Value	B
bFGF-R	-0.18	9.36	-5.60	<0.001	7.89
IGF-I	-0.22	9.58	-4.68	<0.001	3.65
VEGF-D	-0.16	8.85	-3.79	<0.001	0.21
PDGFRA	-0.19	9.72	-2.87	<0.001	-2.71
EGFRvIII	-0.09	14.72	-2.86	<0.001	-2.72
FGF-8B	0.07	9.53	2.33	0.02	-4.05
IGF-I sR	-0.09	12.49	-2.14	0.03	-4.47
FGF9	0.07	8.93	2.10	0.04	-4.54
EGFR	-0.24	8.25	-2.07	0.04	-4.61
EGF	-0.08	9.34	-1.93	0.04	-4.89
FGF-17	0.05	7.62	1.92	0.04	-4.91
FGF-19	-0.23	12.41	-1.86	0.06	-5.02
FGF-5	-0.13	9.20	-1.45	0.15	-5.68
HB-EGF	-0.05	8.04	-1.34	0.18	-5.82
FGF-10	0.11	8.47	1.27	0.21	-5.92
FGF-20	-0.08	8.19	-1.26	0.21	-5.93
FGF-18	0.05	8.17	1.25	0.21	-5.94
FGF-12	0.05	7.44	1.23	0.22	-5.97
FGF-4	0.06	7.58	1.20	0.23	-6.00
VEGF sR2	0.04	13.01	1.17	0.24	-6.03
K-ras	-0.06	9.97	-1.15	0.25	-6.05
FGFR-2	-0.09	8.17	-1.14	0.25	-6.07
PDGF-BB	0.16	14.38	1.14	0.26	-6.07
PDGF-AA	0.12	13.35	1.12	0.26	-6.09
HGF	0.09	10.87	1.08	0.28	-6.13
PDGF Rb	-0.11	11.40	-1.08	0.28	-6.13
FGF7	-0.05	10.05	-1.02	0.31	-6.19
M-CSF R	-0.04	7.26	-0.95	0.34	-6.26
G-CSF	0.08	10.38	0.91	0.36	-6.30
b-NGF	-0.09	9.39	-0.83	0.41	-6.37
G-CSF-R	-0.05	9.96	-0.77	0.44	-6.42
CSF-1	0.02	10.15	0.73	0.47	-6.45
VEGF	0.02	13.09	0.70	0.49	-6.47
bFGF	-0.10	10.68	-0.56	0.57	-6.55
FGFR4	-0.07	10.32	-0.50	0.62	-6.59
GM-CSF	0.04	7.94	0.44	0.66	-6.61
PDGF-CC	-0.04	8.72	-0.43	0.66	-6.62

FGF-6	0.03	8.19	0.40	0.69	-6.63
IGF-II receptor	-0.02	14.96	-0.39	0.69	-6.63
FGF-16	-0.02	10.55	-0.33	0.75	-6.66
FGF-8A	-0.03	8.29	-0.26	0.80	-6.68
VEGF sR3	-0.01	12.97	-0.22	0.82	-6.69
VEGF-C	0.01	9.91	0.19	0.85	-6.69
FGFR-3	-0.01	9.59	-0.17	0.86	-6.70
VEGF121	-0.01	9.26	-0.15	0.88	-6.70
FGF23	0.01	9.03	0.14	0.89	-6.70
RASA1	-0.01	9.59	-0.13	0.90	-6.70

Supplementary Figure S1: Stratification of Control and PCOS women from the database on BMI (BMI ≤29.9 Kg/m^2 and > 29.9 Kg/m^2), then stratified by testosterone into non – hyperandrogenic/ hyperandrogenic (testosterone 1.5nmol/l), then stratified by insulin resistance (HOMA 1.9).

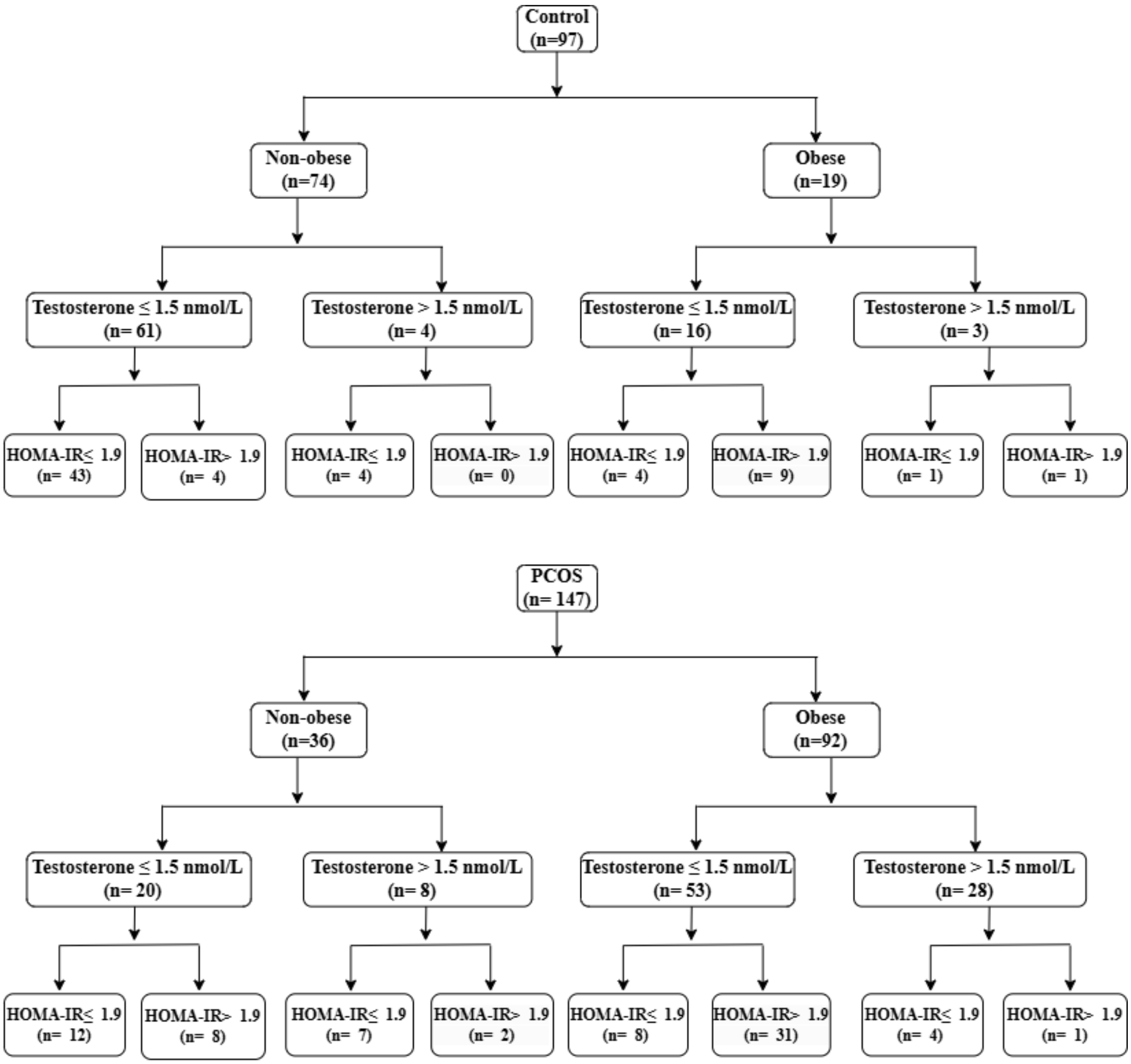

Supplement: Supplementary file 1 [file cells-14-00377-s001.zip › cells-3462454-supplementary.pdf]
